# Enabling and hindering factors of health surveillance assistants’ roles in the provision of contraceptive services in Mangochi, Malawi

**DOI:** 10.1186/s12978-020-0906-3

**Published:** 2020-04-20

**Authors:** Maryse Kok, Madalitso Tolani, Wongani Mtonga, Thom Salamba, Twambilire Mwabungulu, Arnold Munthali, Eefje Smet, Benedict Chinsakaso

**Affiliations:** 1 KIT Royal Tropical Institute, P.O. Box 95001, 1090 HA Amsterdam, the Netherlands; 2 Amref Health Africa, Malawi P.O. Box 30768, Capital City, Lilongwe,; 3 Amref Health Africa, the Netherlands, Schuttersveld 9, 2316 XG Leiden, the Netherlands

**Keywords:** Contraceptive services, Family planning, Community health workers, Malawi

## Abstract

**Background:**

Contraceptive services are essential for promoting people’s health, and economic and social well-being. Despite increased contraceptive use over the past decades, unmet need is still high in Malawi. As a result of task shifting, health surveillance assistants (HSAs), Malawi’s paid community health worker cadre, provide an expanded range of contraceptive services, aimed at increasing access at community level. We conducted a qualitative study to explore enabling and hindering factors of HSAs’ roles in the provision of modern contraceptive services in Mangochi district, Malawi.

**Methods:**

The study involved HSAs and their supervisors, a variety of community members, health workers and policy makers using 34 interviews and 12 focus group discussions. Data were recorded, transcribed, translated, coded and thematically analysed according to a framework that included community-, HSA- and health system-related factors.

**Results:**

HSAs were found to be trusted providers of contraceptive services. At community level, gender norms, decision-making and beliefs about contraceptives were intertwined. They resulted in women using contraceptive services, including those offered by HSAs, in secret. There were misconceptions about contraceptives among both men and women, which were insufficiently addressed by HSAs. Residence and age of HSAs influenced their role in the provision of contraceptive services to (young) community members, whereas sex was not regarded as an enabling or hindering factor. While most community members reported to be satisfied with the quality of HSAs’ services, quality was compromised by a lack of contraceptive supplies and other resources, and limited supportive supervision and training.

**Conclusions:**

HSAs in Mangochi are important contraceptive service providers. Their ability to ensure male involvement, increase access to services for youth and address misconceptions about contraceptives needs improvement. This requires a thorough understanding of socio-cultural norms and improved behavioural change communication competencies, which need to be incorporated in future training under Malawi’s Community Health Strategy.

## Plain English summary

Contraceptive services are essential for promoting people’s health, and economic and social well-being. Despite increased contraceptive use over the past decades, unmet need is still high in Malawi. Health surveillance assistants (HSAs), Malawi’s paid community health worker cadre, provide a range of contraceptive services at community level. We conducted a qualitative study to explore enabling and hindering factors of HSAs’ roles in the provision of modern contraceptive services in Mangochi district. The study involved HSAs and their supervisors, a variety of community members, health workers and policy makers using 34 interviews and 12 focus group discussions. HSAs were found to be trusted providers of contraceptive services. At community level, gender norms, decision-making and beliefs about contraceptives were intertwined. They resulted in women using contraceptive services, including those offered by HSAs, in secret. There were misconceptions about contraceptives among both men and women, which were insufficiently addressed by HSAs. Residence and age of HSAs influenced their role in the provision of contraceptive services to (young) community members. While most community members reported to be satisfied with the quality of HSAs’ services, quality was compromised by lack of contraceptive supplies and other resources, and limited supportive supervision and training. HSAs in Mangochi are important contraceptive service providers. Their ability to ensure male and youth involvement and to address misconceptions about contraceptives needs improvement. This requires a thorough understanding of socio-cultural norms and improved behavioural change communication competencies, which need to be incorporated in future training under Malawi’s Community Health Strategy.

## Background

The Malawi National Sexual and Reproductive Health and Rights Policy stresses the need for increased use of family planning and contraceptive services, pointing at ‘the risks of maternal, infant, and child morbidity and mortality when pregnancies are too early, too many, too late, and too frequent’ [1]. Despite efforts to make contraceptive services accessible to all Malawians, the fertility rate remains high at 4.4 [2]. Knowledge about contraceptives is high, with 98% of women and nearly 100% of men aged 15–49 knowing at least one modern method of contraception. The modern contraceptive prevalence rate (CPR) is 58% among married women (15–49 years) and 43% among sexually active unmarried women aged 15–49 years [2]. Although these percentages show a great increase since 1992 (the modern CPR was 7% by then), the unmet need for family planning among married women (15–49 years) is at 19% and that of sexually active unmarried women (15–49 years) is at 40%. The unmet need is higher for younger and less wealthy women [2].

In 2013, the Government of Malawi embarked on a process to formalize task shifting of some (clinical) tasks to community health workers (CHWs), including health surveillance assistants (HSAs), who constitute the most important paid CHW cadre in Malawi. The tasks that were shifted to HSAs include contraceptive services. Besides awareness raising, health education and referral, their mandate with regard to the provision of contraceptives was expanded from condoms and pills to include the injectable Depo-Provera [3]. HSAs, who are attached to a hospital or health centre but are supposed to spend most of their time in the community, are supervised by senior HSAs and assistant environmental health officers [4]. In relation to the provision of contraceptive services, HSAs link with community midwife assistants and nurses at health facility level for support and referral. In some communities, HSAs link with community-based distribution agents (CBDAs), who are volunteers distributing condoms and pills.

A systematic review on the role of CHWs in the provision of family planning services in low- and middle-income countries has shown that many CHW programmes have increased contraceptive use [5]. In Malawi as well, HSAs have played a critical role in the provision of contraceptives and related services, but there is room for optimization, looking at the CPR especially in rural areas. Similar to global evidence on the optimal design and operation of CHW programmes [6, 7], earlier studies in Malawi stress the importance of improving support to HSAs to conduct their roles and tasks through clear task descriptions, supportive supervision, training, incentives and adequate supplies [3, 4, 8–10]. In addition to health systems factors influencing the ability of HSAs to conduct their roles, community-related factors can significantly influence service provision and utilization of community-based contraceptive services.

Perspectives, preferences and needs of communities with regard to contraceptive services can vary per context. Chipeta et al. (2010) reported that the importance of fertility combined with misconceptions about contraceptives limited their use in Mangochi district [11]. Decision-making on contraceptive use or contraceptive use itself can also be influenced by gendered differences in power. A qualitative study in Ntcheu, Mangochi and Zomba districts found that unsupportive husbands made women to use contraceptive secretly [12]. Husbands’ resistance was related to their desire to exert control over the sexual and reproductive lives of their wives [13]. Yeatman and Trinitapoli (2008) found that contraceptive acceptance differed per denomination in Malawi, with Catholic leaders showing least and Muslim and Pentecostal leaders showing most acceptance of contraceptives. However, the same study found that contraceptive use seemed not to depend upon denomination of women [14].

CHWs’ ability to discuss and respond to communities’ preferences and perceived needs in relation to sexual and reproductive health, including contraceptive services, is instrumental to increase the use of these services. CHWs in Uganda reported to find it difficult to approach clients because of the community stigma attached to family planning; instead they would wait for clients to approach them and ask for the support of community leaders in conducting their awareness activities [15]. Sambakunsi et al. (2015) reported that HSAs in Malawi faced problems with the provision of HIV/AIDS services, because of apostolic faith people not accepting any health services [16]. In a study on the promotion of male involvement in maternal health in southern Malawi, professional health workers reported that HSAs were trusted promoters of male involvement because of their residence in the community, which made them well placed to convey messages in a socio-culturally accepted manner [17].

**Fig. 1:**
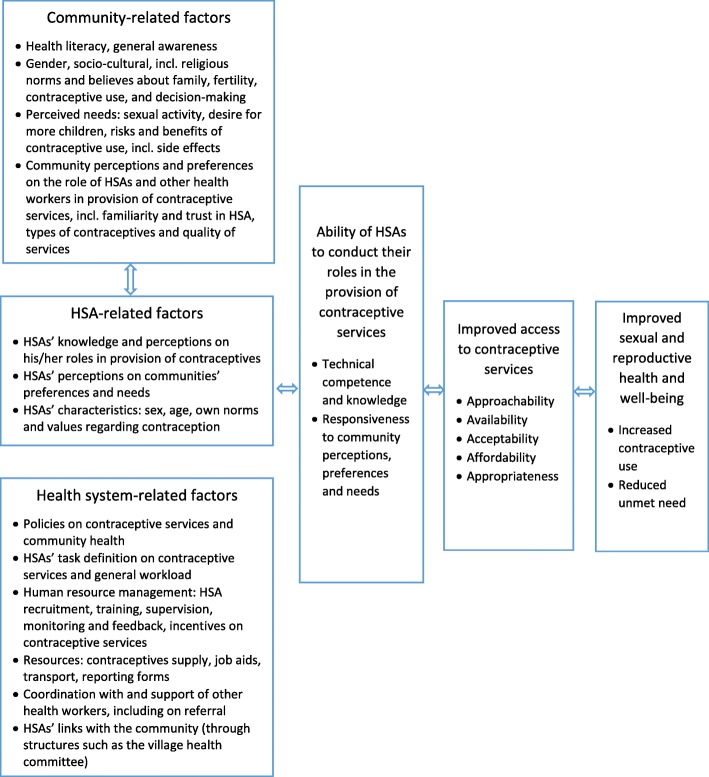
Conceptual framework on the factors that could influence HSAs’ ability to conduct their roles and tasks in the provision of contraceptive services in Malawi

Following the above, the provision of contraceptive services can be influenced by the way CHWs are trained, supplied and supervised (factors related to the health system), but also by perspectives, preferences and needs of community members that are related to gender and socio-cultural norms, including religion (community factors). In addition, CHWs’ personal characteristics, perceptions and preferences can have a bearing on their ability to perform their roles and tasks. Figure 1 provides a conceptual framework on the factors that could influence HSAs’ ability to conduct their roles and tasks in the provision of contraceptive services in Malawi. The framework is based on general frameworks on CHW performance [18–20], access to care [21] and the above presented evidence on communities’ preferences and perceived needs in relation to sexual and reproductive health [11–17].

While it is generally supported that HSAs, being CHWs, can improve access to services because of their proximity to the community, the complexity of different factors that influence HSAs’ ability to conduct their roles and tasks in the provision of contraceptive services is not yet well understood. We conducted a qualitative study to explore enabling and hindering factors of HSAs’ roles in the provision of modern contraceptive services in Mangochi district, Malawi. The ultimate goal was to provide information for policy makers and programme managers on how to optimize HSAs’ role, to ultimately contribute to increased contraceptive use in Malawi.

## Methods

### The context

We conducted a qualitative study in three Traditional Authorities (TAs) of Mangochi district in southern Malawi. Mangochi district was selected because Amref Health Africa, the convener of this study, is implementing an advocacy campaign for increased support and recognition of CHWs in Mangochi^1^. The diversity of the district population allowed comparing and contrasting on findings across tribes and religions. Mangochi has a population of 1,091,666 and a fertility rate of 5.3 (against the national figure of 4.4) and a modern CPR of 31% among married women aged 15–49 years (against the national figure of 58%). The unmet need for family planning among the same group is 30%, against 19% nationwide. 36.7% of young females aged 15–19 have begun childbearing [2]. Seventy percent (70%) of the population are Yaos while other tribes (e.g. Chewa, Tumbuka, Mhlomwe) consist of 30%. Farming is the major source of income in Mangochi and the fishing industry provides income to approximately 5% of the population. The district has 43 health facilities consisting of four hospitals, 29 health centres and 10 dispensaries. Of the 43 health facilities, 25 are government owned. Among the other 18 health facilities, 15 are owned by the Christian Health Association of Malawi (CHAM) [22] and three by private practitioners. All health facilities cater for a total of 1254 villages which are administratively grouped into 13 TAs under the overall authority of the District Council [23]. The district has 487 HSAs, meaning that on average one HSA serves a population of 2242, which is much above the national guideline of one HSA per 1000 population.

This study took place in TA Makanjira and Katuli, which are predominantly Yao, and in TA Namkumba, where Chewa and, to a less extent, other tribes are living. In these TAs, the data collection took place in the catchment areas of three health facilities; Lugola health centre (which is a CHAM Catholic facility) in TA Makanjira; and Katuli and Namkumba Health Centres (government facilities).

### Study participants and data collection

Data collection took place at three levels: at the community, district and national level, using interviews and focus group discussions (FGDs). In each TA, at the community level, data were collected from individual women aged 15 to 49 (married and unmarried) who either used, stopped using or never used modern contraceptives. In two TAs, semi-structure interviews (SSIs) with men were conducted. All SSIs aimed to gain insights into personal perspectives about the role of HSAs in the provision of contraceptive services and enabling or hindering factors with regard to the use of these services. We also conducted SSIs with trained HSAs, community midwife assistants (in all TAs) and a CBDA (in only one TA). In addition, in each TA, FGDs with married and unmarried community members and with HSAs were conducted. Furthermore, key informant interviews (KIIs) with assistant environmental health officers (AEHOs) were conducted in all TAs; and KIIs with influential individuals, such as community leaders, were conducted in two TAs. These FGDs and KIIs also focused on the role of HSAs in the provision of contraceptive services. At district level, we conducted KIIs with CHW supervisors at the district health office and representatives from non-governmental organizations (NGOs) working in the area of family planning. These interviews aimed to provide in-depth insights from the provider and health system perspective. Lastly, KIIs were conducted at the national level with relevant authorities within the Ministry of Health and a national level NGO (Table 1).

**Table 1:** Overview of methods and study participants

Method	Study participants	Number of participants and specifications
SSIs	Women (15–49 years)	1 unmarried using contraceptives 1 married stopped using contraceptives 1 unmarried stopped using contraceptives 2 married never used contraceptives 1 unmarried never used contraceptives
	Men	2 married from Yao and Chewa tribe
	HSAs	2 trained male HSAs, government facilities 2 trained female HSAs, government facilities 1 trained female HSA, CHAM facility
	CMAs	3, from each of the 3 TAs
	CBDAs	1, from one of the 3 TAs
FGDs	Female community members	3 FGDs, unmarried 15–18 yrs. (1 from each TA); 20 participants 3 FGDs, married 19+ yrs. (1 from each TA); 22 participants
	Male community members	1 FGD, unmarried 15–18 yrs.; 7 participants 3 FGDs of married 19+ yrs.; 22 participants
	HSAs	3 FGDs (1 from each TAs); 18 participants
KIIs	Community Level	2 traditional leaders (TAs) 3 religious leaders (1 Muslim, 1 Catholic and 1 Christian other) 3 AEHOs (1 from each of the TAs) 1 representatives of a safe motherhood group 1 female initiator
	District Level	1 representative from the environmental health department 1 representative from the nursing department 1 family planning coordinator 1 NGO representative
	National level	1 representative of in the Reproductive Health Unit 1 representative of the Community Health Section 1 representative of the White Ribbon Alliance

Purposive sampling was used in the selection of participants to ensure that people of different tribes and religions were selected, as well as users and non-users of contraceptives. All HSAs of the three included health centres were selected for this study, only those who went outside Mangochi (for example for training) did not participate. Senior HSAs at the selected health centres assisted the research team in the sampling and recruitment of community members.

Eight trained research assistants, supervised by two Amref staff, collected the data in January 2019 for 2 weeks. The team made use of various topic guides, which were based on the analytical framework, tailored to the method and participant group, and pre-tested in TA Chowe in Mangochi, after which they were slightly adjusted and finalized. At community level, the interviews and FGDs were conducted in Chichewa, and at times partly in Yao. Interviews at district and national level took place in English. During data collection, four debriefings were held with all research assistants to discuss progress, preliminary findings and adjust sampling strategies where needed.

### Data analysis and validation

Interviews and FGDs were digitally recorded and transcribed in English. The analytical framework formed the basis for the development of a coding framework, in which emerging themes were added. Transcripts were coded in Nvivo 11 and data were further analysed, ‘charted’ in themes and subthemes and summarised in narratives. While the coding was done by one researcher, the full data analysis was conducted in a team of researchers, Amref staff and one research assistant. Preliminary findings from this study were validated with the Mangochi District Research Committee and the Health Sub-committee of the District Executive Council of Mangochi.

### Ethical considerations

The National Health Science Research Committee provided ethical approval for this study. In addition, the District Health Management Research Team gave their approval to conduct this study. All study participants signed an informed consent form. For participants who were less than 18 years old, consent was sought from their parents or guardians prior to the interviews.

## Results

Table 1 summarizes the number of study participants and their characteristics per study method.

We present our study findings according to the three main factors that influence HSAs’ roles in the provision of contraceptive services: community-, HSA- and health system-related factors. Within those categories, the major emerging themes are presented (Table 2).

**Table 2:** Overview of factors that influenced HSAs’ roles in the provision of contraceptive services

Major emerging theme	Summary of findings	How it influenced HSAs’ roles in the provision of contraceptive services
*Community-related factors*		
General awareness and access of contraceptive services	Community members were generally aware of possible advantages of modern contraceptives, and where to access them.	This enabled HSAs’ roles in the provision of contraceptive services, as people had basic knowledge about these services and some would actively approach HSAs.
Cultural and gender norms	Cultural norms around the importance of having many children resulted in a low perceived need for contraceptives, especially among men.	This hindered HSAs’ roles in the provision of contraceptive services, as HSAs seemed not to involve men in awareness raising activities.
	Gender norms around the importance of attractiveness of women, especially in a culture where polygamy is there, resulted in some women perceiving a need for contraceptives.	The exact influence on HSAs’ roles in the provision of contraceptive services is unclear, however women’s perceived need for contraceptives fitted with the women-centred focus of HSAs’ contraceptive service provision.
	Gender norms around women’s responsibility to childcare and contraception led them to often secretly decide on contraceptive use.	This did not enable or hinder HSAs’ roles in the provision of contraceptive services. HSAs did not encounter a lot of direct problems while secretly providing services.
Misconceptions	Misconceptions about contraceptives negatively influenced the perceived need for and decision-making on contraceptive use.	This hindered HSAs’ roles in the provision of contraceptive services, as HSAs seemed not able to address these misconceptions.
Religion	A few religions did not officially support contraceptive use, but seemed to silently tolerate it.	This did not directly hinder HSAs’ roles in the provision of contraceptive services, but HSAs got limited support of some religious leaders during awareness raising.
Perceived quality of HSA-provided care	The majority of the study participants perceived the quality of care provided by HSAs as good.	This enabled HSAs’ roles in the provision of contraceptive services, as they were approached and supported by community members.
*HSA-related factors*		
HSAs’ knowledge and role perception	HSAs were well aware of their roles in the provision of contraceptive services, the importance of these roles and their position regarding other health workers.	This enabled HSAs’ roles in the provision of contraceptive services, as they transferred their knowledge to community members and knew when to refer if needed.
HSAs’ norms and values on contraception	For the majority of the HSAs, their own norms and values on contraception were in line with their expected roles.	This enabled HSAs’ roles in the provision of contraceptive services.
HSAs’ characteristics	Age, sex and origin of HSAs did not significantly influence their relationships with community members.	This did not enable or hinder HSAs’ roles in the provision of contraceptive services. However, older HSAs might be less able to serve youth.
	Community members preferred HSAs residing in their area.	This hindered HSAs’ roles in the provision of contraceptive services, because it was impossible for them to reside in all their catchment areas as they had large areas to cover.
*Health system factors*		
Policies and related resources	Inadequate policy dissemination and resources led to challenges in policy implementation.	This generally hindered HSAs’ roles in the provision of contraceptive services.
	NGOs had a large role in (facilitation of) the provision of contraceptive services.	This enabled HSAs’ roles in the provision of contraceptives services, through provision of training and supplies, however, it hindered their roles because of challenges regarding coordination and perceived inequity of support between areas.
HSAs’ training	HSAs’ training in the provision of contraceptive services was dependent on partners and therefore, only half of them were trained.	While this enabled trained HSAs in providing contraceptive services, the differences between trained and untrained HSAs led to confusion or mistrust in the community, which hindered HSAs’ roles in the provision of contraceptive services.
HSAs’ supervision and monitoring	There was a disconnect between the environmental health and nursing department with regard to supervision of HSAs.	This hindered HSAs’ roles in the provision of contraceptive services, as their direct supervisors, AEHOs, felt disconnected to the family planning programme and thus provided limited supervision.
	Supervision was more administrative than supportive in nature.	This hindered HSAs’ roles in the provision of contraceptive services, as feedback did not focus on quality of care and client satisfaction.
Contraceptive supply	There was erratic supply of contraceptives at health centre and community level.	This hindered HSAs’ roles in the provision of contraceptive services, partly because of dissatisfied clients.
HSAs’ working relationships with others	HSAs had generally good working relationships with health professionals.	This enabled HSAs’ roles in the provision of contraceptives services, partly through effective referral.
	Community groups and traditional/ religious leaders sometimes assisted HSAs by provision of platforms for awareness raising.	This enabled HSAs’ roles in the provision of contraceptives services, however, a more active role of traditional leaders would further enable HSAs’ work.
Other resources	Stationary supply was constrained, and transport remained a challenge for HSAs, although they were provided with pushbikes.	This hindered HSAs’ roles in the provision of contraceptive services, specifically in relation to quality of care and provision of services in hard-to-reach areas.

### Community-related factors that influence HSAs’ roles in the provision of contraceptive services

#### General awareness and access of contraceptive services

All study participants were aware of different types of modern contraceptives and their (possible) advantages – of which economic advantages for the family were most mentioned. However, knowledge did not always translate into behaviour and thus contraceptive use. The majority of the study participants said that HSAs are the primary and preferred providers of contraceptives, because they are trusted and easily accessible. Nurses and Banja la Mtsogolo (an NGO) were said to provide long-lasting contraceptives, and in Namkumba, CBDAs were mentioned. In Makanjira, at the Catholic CHAM facility, contraceptives were not provided. However, many female community members and all HSAs reported that contraceptives were well available and provided by HSAs.

#### Cultural and gender norms

Some study participants in Katuli and Makanjira, where it is predominantly Yao, expressed that culturally, men have esteem among their peers when they have many children; which makes many of them against the use of contraceptives. The Yao culture is predominantly Muslim and accepts polygamy. Some women claimed not to use contraceptives, because they wanted to “give their husbands more children”, which meant they got more attention from their husbands. There were other women who said that they used contraceptives to remain attractive for their husbands thereby avoiding them taking a younger second wife.

“A woman who doesn’t follow family planning is always busy with children at home and poverty is always on her because of the many children. You also appear attractive to your husband when you practice family planning because you don’t get old quickly.” (FGD, married females 19+ yrs, Makanjira)

Both male and female community participants agreed that child upbringing is more the woman’s responsibility than the man’s. After having several children, a man could easily leave his wife to go to South Africa for work, or marry another woman. The women would be left with the responsibility of taking care of the children. Because of this, decision-making about contraceptive use was seen as a women’s issue, though it was influenced by men. When husbands did not support contraceptive use, most women preferred to use Depo-Provera. Some had two health passports and visited the HSA at night, to secretly receive the injection. HSAs assisted women in secret use of contraception and were, in their awareness raising activities, more focused on women than men. There were not many accounts of men feeling uncomfortable about confidential arrangements between HSAs and their wives, but one married man in Katuli said that it was a problem to him. Some participants said that secret contraceptive use could lead to divorce when the husband found out.

The study revealed that sex was seen as for those who are married, which meant that some people in the community saw young people who access contraceptives, especially girls, as promiscuous. Young female participants in an FGD (15–18 years) reported that the taboo made many young people to hide contraceptive (including condom) use from their parents. While HSAs kept young people’s contraceptive use confidential, they seemed not particularly knowledgeable in how to face possible resistance when covering the subject of prevention of teenage pregnancy with parents.

#### Misconceptions

Community members, in particular adult and young men, had many misconceptions about contraceptives. Contraceptives would reduce sexual desire and performance and cause diseases, body deformations, or barrenness. Some female study participants argued that side effects, such as nausea and heavy menstruations caused by pills or Norplant, or cease of menstruation while using Depo Provera made them stop using contraceptives. Misconceptions seemed to emerge after talking to people in the community who explained what they had experienced themselves, or explained what they had heard from others.

“The contraceptives have weakened our men. They could sleep with us three times a day, but not now, they can’t perform…” (FGD, married females 19+yrs, Makanjira)

#### Religion

The majority of the study participants said that religion did not influence people’s need for and decision-making on contraceptives, except that Catholics and Bible believers do not support the use of modern contraceptives, because of the importance of reproduction and avoiding the sin of ‘killing the child’. While these churches did not support contraceptive use, they seemed to tolerate it silently.

“I am a church leader and the bible says we should multiply like the sand. For the people to go to the hospital for family planning methods we cannot condone that, but since the way the organizations are lined up these days, we encourage some people that the way the world is these days, it’s good for them to go to the hospital and follow family planning, but according to the bible, it is unacceptable.” (KII, Christian religious leader, Namkumba)

#### Perceived quality of HSA-provided care

The majority of the study participants perceived the quality of care provided by HSAs as good, as they take time to provide counselling. Despite this, women were dissatisfied if contraceptives were out-of-stock, or if untrained HSAs needed to refer them to trained HSAs in case they needed Depo-Provera. A few women thought that quality of care could increase if HSAs would be equipped with BP-machines and pregnancy tests. A few female and male participants thought that HSAs should improve on explaining side effects and addressing misconceptions.

### HSA-related factors that influence HSAs’ roles in the provision of contraceptive services

#### HSAs’ knowledge and role perception

HSAs were clear about their roles in the provision of contraceptive services. They used a checklist that assists in deciding which contraceptive methods are best for particular clients. They referred to health professionals in case clients faced problems with contraceptive use, when short-term contraceptives were out of stock, or when clients preferred long-term contraceptives. A few HSAs reported to supervise CBDAs, which mainly meant supplying them with pills and condoms. HSAs stressed confidentiality, politeness, being a good example and good communication skills as important in their job.

“As HSAs we must make sure we are polite with the people, we shouldn’t think that we are more knowledgeable just because we have the positions. Because if we don’t, they will not get the contraceptives and they hate you. When you are polite with them they even help in encouraging others to come to you and get the contraceptives.” (FGD, HSAs, Makanjira).

One HSA in Kutuli reported that HSAs are a bridge between clients and health professionals: in case women would like to start long-term contraceptives, they fear going to a doctor or nurse and would use the HSA to approach these professionals. All HSAs seemed to support the economic and health-related advantages of contraceptive use. HSAs explained these advantages, mainly to women, to encourage the use of contraceptives.

#### HSAs’ norms and values on contraception

While the majority of the HSAs felt comfortable providing contraceptive to the youth, one HSA in Katuli mentioned young people’s ‘abstinence stage’, implying he was not comfortable with providing contraceptives to youth. The religion of the HSAs did not influence their opinions nor ability to provide contraceptive services. There was only one account of a married women who reported that an HSA asked her irrelevant questions when she came for contraceptives: whether she was married, and whether her husband was around.

#### HSAs’ characteristics

Many youth indicated that the age of the HSA makes no difference to them, their training and experience was found more important. However, the CMAs in Katuli and Namkumba and some youth in Namkumba felt that the age gap between the youth and the HSAs may put off the youth to obtain contraceptives. Therefore, more young CBDAs were recommended. The majority of the participants indicated that the sex of the HSA has no effect on the delivery of and access to contraceptive services. Here as well, professionalism was considered most important. One unmarried female FGD participant from Namkumba indicated that she preferred male HSAs, because she had heard that female HSAs can be brutal, while male HSAs “know the pain women pass through” and are more considerate.

Some HSAs were said to reside in the community, some were living closer to the trading centre, where the health centre was located. The latter were less accessible to people in the community. HSAs as well as community members indicated that it is not possible to reside in all communities they serve, simply because they cover four to five villages each and have a heavy workload.

“They [the government] … should increase the human resource of HSAs, because one can be overseeing four village development committees and 10 villages so they should at least be in pairs per catchment area not one as it is.” (SII, married male Yao, Katuli)

Most community-level participants in Katuli believed that HSAs who live in their community of service build trusting relationships with community members, hence they may assist the community better. None of the HSAs in the study areas originally came from the area they served. The majority of the community-level participants did not believe that the origin of the HSAs affects (quality of) service delivery. A few community members in Makanjira suggested that having someone originating from the community might be a disadvantage, as some HSAs might start gossiping about women taking contraceptives. However, some thought that not having an indigene is a disadvantage in relation to earning respect of the people, and being conversant with the language and culture in the area. HSAs from inside the community may also be good role models. One young woman referred to the downside of transferring HSAs (which currently happens), leading to a lack of continuity.

### Health system factors that influence HSAs’ roles in the provision of contraceptive services

#### Policies and related resources

National and district-level key informants mentioned inadequate dissemination of family planning policies as one of the challenges in the implementation of these policies, besides financial constraints. While district-level informants indicated that NGOs enabled the delivery of contraceptive services to the community, national-level informants pointed towards problems with coordination of these partners.

#### HSAs’ training

About half of the HSAs from the three study areas were trained in the provision of Depo Provera. As reported, this sometimes caused confusion or mistrust at community level, when untrained HSAs had to refer to trained HSAs or other health workers.

“Number 6 [referring to a colleague HSA] and I come from very far villages, and when we tell the people to come here at the hospital to get the Depo, they find the nurse busy and they go back home, 26 to 28 kilometres without any help. If we were trained, we would help them in the outreach clinics.” (FGD, HSAs, Namkumba)

The training of HSAs seemed to solely depend on the support of NGOs. While it was clear that these partners considered the context – for example, most HSAs in Makanjira, where the Catholic CHAM facility is located, and all HSAs serving hard-to-reach areas, were trained – one HSA reported that only active HSAs were selected for the training. This created feelings of inequity among HSAs, also because trained HSAs were entitled to bikes and other supporting materials, and earned more respect among community members than untrained HSAs.

#### HSAs’ supervision and monitoring

A national-level key informant reported fears regarding inadequate reporting lines between HSAs and nurses, as contraceptive services fall under the nursing department. The issue was confirmed at district level, where AEHOs felt left out on issues to do with the family planning programme, and where the nursing department reported to be not enough in charge of HSAs’ roles in the provision of contraceptive services. There seemed to be a disconnect between the nursing and environmental health departments. AEHOs were not invited for trainings of HSAs with regard to the provision of contraceptive services. Therefore, not all AEHOs felt responsible for supervising HSAs on contraceptive services.

“…the linkage between the family planning coordinator and the supervisor of the HSA should be good. Because some time at some point you just hear the HSAs have gone for training and you wonder why there is no linkage. So at some point as a supervisor you say I am not important in this programme so you start neglecting or not supervising it, it’s like you are not part of it.” (KII, AEHO)

Furthermore, AEHOs seemed to conduct ad-hoc supervision, mainly reacting to problems (e.g. contraceptive stock-out) and questions from HSAs, and ensuring that all necessary reports were submitted to the district health office. Provided feedback focused on utilization figures from the reports and not on quality of care or client satisfaction. At community level, CMAs are also supposed to play a role in the supervision of HSAs, however, this did not take place, as CMAs are based at facility level because of staff shortages.

Two HSAs from different areas reported that tally sheets and reporting forms were not available and that HSAs used plain paper, which was confirmed by the district level family planning coordinator. Some HSAs indicated that monitoring forms should have more focus on following clients over time, so that HSAs are notified when women are due for a new injection. This would improve quality of care.

#### Contraceptive supply

The study revealed that there was erratic supply of contraceptives, with many participants elaborating that this is usually for the most popular methods like Depo Provera.

“The methods are not there, it’s now six months without contraceptives and many [people] like the Depo but it’s not there. And now the people have lost hope in family planning.” (FGD, HSAs, Lugola)

When there were stock outs, women were encouraged to change contraceptive methods, which impeded their choice and sometimes led to stopping contraceptive use and mistrust against HSAs or other health workers. The claim of erratic supply of contraceptives was more pronounced at community level than at district and national level. The study found that stock-outs at health centre level related to a problem in the supply system. It was reported that district hospitals, as well as health centres, can only order according to the number of commodities used in the last consignment. This means that if from the start more contraceptives were needed than were supplied, or if demand increases because of active awareness raising, the next supply is still “stuck” at an inadequate number of contraceptives.

HSAs are supposed to get their contraceptive supplies at health centre level. Due to stock-outs at the health centre, they often used their own money to go to the district hospital to try to obtain contraceptives. Some HSAs indicated that NGOs such as Banja la Mtsogolo and the Family Planning Association of Malawi (FPAM) compensated supply of contraceptives when they were out-of-stock in public facilities.

#### HSAs’ working relationships with others

HSAs generally had good working relationships with health professionals at the health centre level. Referral was conducted orally. CMAs joined HSAs in outreach services now and then. Besides assisting with contraceptive supply, FPAM and Banja la Mtsogolo conducted awareness raising sessions in the communities, which was helpful for HSAs. However, in an FGD with HSAs in Katuli, it was reported that some partners and senior staff from the district level come along with HSAs from the district hospital or other areas to assist in the provision of contraceptive services in their villages, without the HSAs from the area knowing this or being involved. This brought confusion among communities and demotivation among the HSAs from the area. From the side of the community, women groups, youth clubs, village development committees and village health committees, assisted HSAs mainly through being a platform for awareness raising. There were mixed reports on support from traditional and religious leaders.

#### Other resources

While the supply of stationary was problematic, all HSAs in the studied areas had bikes and uniforms. An AEHO from Katuli explained that the provision of other resources, such as t-shirts, umbrellas, gumboots or bags, is dependent upon NGOs. Therefore, there can be variations in what the HSAs receive between areas in Mangochi. The HSAs in Namumba purchased motorbikes as part of an NGO-led programme that included performance-based incentives. The bikes, however, were not used because of a lack of fuel. As mentioned above, the study revealed that HSAs who were trained in the provision of contraceptive services got an extra bicycle (from the partner who conducted the training).

“The bicycles that we receive for normal work are the ones that we use. As for our colleagues that are providers for family planning, they are also given special bikes for family planning; only those who inject Depo.” (SSI, male HSA, Namkumba)

This inequity, the fact that the general bikes were distributed 5 years ago and mostly broken, and the large distances between villages making push bikes not to suffice, left HSAs dissatisfied with their mode of transport and limited their ability to conduct their roles in the provision of contraceptive services.

## Discussion

This study aimed to find out which factors influence the ability of HSAs, the largest group of primary health care providers in Malawi, to conduct their roles in the provision of contraceptive services in Mangochi. The factors have been categorized into community, HSA and health system-related factors. It should however be acknowledged that the different factors influence each other, because health systems are complex social institutions [24].

At the level of the community, gender norms, decision-making and beliefs about contraceptives were intertwined. Family planning was seen as a women’s issue, but men have a say in it, being the heads of families, as found by other studies [25, 26]. While the Malawi Demographic and Health Survey found that 80% of married women who used family planning reported that using contraception is usually a joint decision between the wife and her husband [2], in our study areas, many married women seemed to use contraceptives without their husbands’ knowledge. This was the case for Yao, Chewa as well as other tribes. A qualitative study of Kaneka and Mturi (2015) in Ntcheu, Mangochi and Zomba districts found that reasons for secret use of contraceptives by young married women were: the fear of being abandoned by the husband (because of not looking attractive as a result of frequent child bearing), being in a hostile or unstable marriage, lack of communication between spouses, lack of resources to raise children, uncertainty over husbands’ return dates from South Africa and safeguarding women’s health [12]. Many of these reasons were also found in our study, where the lack of resources to raise children was most profound and, as Kaneka and Mturi (2015) indicate, could be related to Mangochi being matrilineal, where the women and her family are responsible for raising the children.

While some male participants reported to support contraceptive use, others did not. This seemed related to the norm that men with many children gain more respect, but also to misconceptions about contraceptives, in particular that they reduce men’s sexual performance [11, 27]. Kaneka and Mturi (2017), in their further analysis regarding the above-mentioned study, found that husbands’ resistance to contraceptive use was related to their desire to exert control over the sexual and reproductive lives of their wives. Non-use of contraceptives would reduce women’s extramarital sexual activities and make them less attractive to other men because of frequent child bearing [13].

It could be argued that the limited involvement of men in the (final) decision for contraceptive use influences misconceptions, in particular around Depo Provera, which is the most preferred method in case of secret use [28]. We found that HSAs mainly target women with awareness raising and assisted them with using contraceptives secretly, which might contribute to persisting misconceptions. HSAs seemed to insufficiently involve male and address misconceptions and potential side effects of contraceptives. To address this, training in communication about (sensitive) topics – such as misconceptions, women’s secret use and contraceptive provision to unmarried youth – and behavioural change competencies is needed. This could be taken up in the future one-year HSA training, as outlined in the Community Health Strategy [29].

While traditional leadership in maternal, new-born and child programming seems to strengthen in Malawi [30], we found that they could play a more active role in supporting HSAs in providing contraceptive services. Contraceptive use did not depend upon denomination [14], however, religious leaders seemed ‘split’ between following the values of their religion, implying contraceptives should not be used, versus accepting people to use contraceptive because of the realities of today. The latter provides a basis for further interactions between them, HSAs and other health workers in their efforts to increase contraceptive use.

With regard to HSA-related factors, from the perspective of community members, the HSAs’ residence seemed to influence their ability to conduct their roles, whereas their tribe, sex and place of origin did not. When HSAs reside in their catchment area, trusting relationships have proven to contribute to better performance [4]. The Government of Malawi intends to increase the number of HSAs over the coming years and recognizes the importance of HSAs residing in their catchment area [29]. The average age of the HSAs we spoke to was 40 years. Although many youth indicated that the age of the HSA makes no difference to them, some study participants mentioned that young people prefer to be assisted by younger health workers. Research shows that young people like to discuss issues around sexual and reproductive health with peers or young role models [31, 32], but young CBDAs seemed to be scarce in the three study areas. Further research should explore collaboration between HSAs and this voluntary cadre in providing youth-friendly services.

Most participants spoke about trust and confidentiality when asked about the quality of HSAs’ services. This signifies the role of HSAs regarding the provision of contraceptive services. The Malawi Service Provision Assessment Survey 2014–15 concluded that only 4% of all observed family planning consultations (conducted by different types of health workers) had all elements of reproductive history (including current pregnancy status) and none of the consultations for new clients had all risk history assessed. Weight and BP were mostly checked [33]. In this study, we found that HSAs do not have BP machines, but a CMA at health centre level also complained about a lack of BP machines and a lack of time for testing BP. Therefore, the quality of care seemed compromised for women who start using contraceptives. HSAs do use a checklist for deciding which contraceptive is suitable for a woman, but it was unclear to what extent this checklist was consistently followed, as supervision was irregular and focusing on health care utilization data instead of quality of care. In 2010, after a pilot on the distribution of injectable contraceptives by HSAs, it was concluded that most but not all HSAs followed all procedures for the safe provision of injectables, and that more frequent supervision focused on quality aspects was recommended [34]. Inadequate supervision, however, seemed one of the main health system-related factors that hindered HSAs’ roles in the provision of contraceptive services. Many studies have pointed towards the importance of supportive supervision of CHWs to enhance their motivation and performance [35]. We found a disconnect between the environmental health department, under which HSAs officially fall, and the nursing department, which is responsible for the family planning programme. A lack of supportive supervision cannot only lead to demotivation and underperformance of HSAs, it could also put HSAs and their supervisors in a difficult position when things go wrong, and clients accuse health workers of malpractice.

We found that only half of the HSAs were trained with regard to contraceptive services. Trained and untrained HSAs had different access to resources, which triggered demotivation. Besides this, it limited communities’ access to Depo Provera, the most popular contraceptive. It is clear that this situation evolved because of the dependency of the Malawi Government on their partners to conduct these trainings, for which HSAs serving hard-to-reach areas were prioritized. A stronger coordinating role of the Malawian Government towards aligned policy and programming on HSAs would be recommended.

The majority of study participants indicated that there are often stock-outs of contraceptives at the health centre and community level. This led to limited choice on the type of contraceptive to be used, which is a violation of human rights [36]. It seemed that stock-outs were not always a result of a lack of commodities, but rather a result of a problem in the supply chain [37]. Exploration of the possibility to change the system from ordering based on use (‘pull system’) to ordering based on forecasted needs ‘push system’ could be considered.

Strengths of this study are that data analysis and discussion of findings took place in a multi-disciplinary group of researchers, NGO and government staff and that triangulation of findings from different methods and sources took place. The data collection team put much effort in selecting study participants with different characteristics. However, it needs to be noticed that most participants lived relatively close to the health centre. Furthermore, it needs to be acknowledged that the team spoke to relatively high-educated women. We can therefore assume that the situation in more hard-to-reach areas might be more problematic than is presented in this paper. While the study included various (tribal) groups in Mangochi, the study design does not allow for generalizability of findings to other districts in Malawi. However, the above comparisons with the literature do show similarities across contexts in Malawi.

## Conclusions

This study identified both enablers and barriers of HSAs’ roles in the provision of contraceptive services in Mangochi, Malawi. HSAs were found to be trusted providers of contraceptive services at the community level, however, there is room for improvement in relation to their ability to ensure male and youth involvement and address misconceptions about contraceptives. This should be taken up in the future one-year HSA training, as announced in the Community Health Strategy. Policy implementation with regard to HSAs’ residence, training, supportive supervision and the contraceptive supply chain needs further attention. Joint supportive supervision by the environmental health and nursing department and improved supply of contraceptives can improve HSAs’ roles in the provision of contractive services, which can further enhance sexual and reproductive health and rights in Malawi.

## Abbreviations

AEHO: Assistant environmental health officer
CBDA: Community-based distribution agent
CHAM: Christian health association of Malawi
CHW: Community health worker
CMA: Community midwife assistant
CPR: Contraceptive prevalence rate
FGD: Focus group discussion
HSA: Health surveillance assistant
KII: Key informant interview
NGO: Non-governmental organization
SSI: Semi-structured interview
TA: Traditional authority

## Footnotes

1 Amref Health Africa implements this programme in Ntchisi and Chitipa Districts as well.
